# (μ-Diphenyl­phosphanido-κ^2^ *P*:*P*′)bis­[2,2′-(pyridine-2,6-diyl)diphenyl-κ^3^ *C* ^1^,*N*,*C* ^1′^)gold(III)] perchlorate acetonitrile solvate

**DOI:** 10.1107/S1600536808024537

**Published:** 2008-08-06

**Authors:** Xin-Sheng Li, Juan Mo, Su-Mei Zhang, Li Yuan, Jian-Hua Liu

**Affiliations:** a College of Animal Husbandry and Veterinary Studies, Henan Agricultural University, Zhengzhou, Henan Province 450002, People’s Republic of China

## Abstract

The title complex, [Au_2_(C_17_H_11_N)_2_(C_12_H_10_P)]ClO_4_·C_2_H_3_N, contains two Au^III^ atoms bridged by a diphenyl­phosphanide ligand. Each Au atom is in a square-planar environment coordinated by diphenyl­phosphanide and 2,6-diphenyl­pyridine ligands. There are weak π–π stacking inter­actions between neighbouring mol­ecules (the inter­planar separations between two neighbouring dpp units are 3.40 and 3.57 Å). The intra­molecular Au⋯Au separation is 3.788 (5) Å. The crystal structure shows weak inter­molecular C—H⋯O and C—H⋯N hydrogen bonds involving an O atom of the perchlorate counter-ion and the N atom of the acetonitrile solvent mol­ecule, respectively.

## Related literature

For related literature, see: Goshe *et al.* (2003 ▶); Kui *et al.* (2006 ▶); Li *et al.* (2006 ▶); Lu *et al.* (2004 ▶); Wong *et al.* (1998 ▶); Yam *et al.* (2002 ▶). 
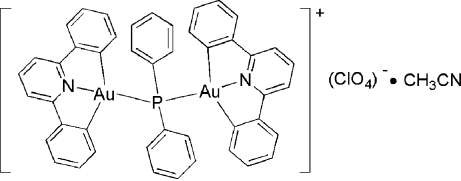


## Experimental

### 

#### Crystal data

[Au_2_(C_17_H_11_N)_2_(C_12_H_10_P)]ClO_4_·C_2_H_3_N
*M* *_r_* = 1178.14
Monoclinic, 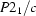

*a* = 10.0612 (16) Å
*b* = 13.526 (2) Å
*c* = 29.640 (5) Å
β = 98.828 (4)°
*V* = 3985.9 (11) Å^3^
*Z* = 4
Mo *K*α radiation
μ = 7.51 mm^−1^
*T* = 113 (2) K
0.32 × 0.22 × 0.20 mm

#### Data collection

Bruker SMART CCD area-detector diffractometer
Absorption correction: multi-scan (*SADABS*; Sheldrick, 1996 ▶) *T*_min_ = 0.147, *T*_max_ = 0.221
38570 measured reflections
10221 independent reflections
8923 reflections with *I* > 2σ(*I*)
*R*_int_ = 0.039

#### Refinement

*R*[*F* ^2^ > 2σ(*F* ^2^)] = 0.028
*wR*(*F* ^2^) = 0.057
*S* = 1.04
10221 reflections
534 parameters
H-atom parameters constrained
Δρ_max_ = 1.52 e Å^−3^
Δρ_min_ = −1.55 e Å^−3^

### 

Data collection: *SMART* (Bruker, 1997 ▶); cell refinement: *SAINT* (Bruker, 1997 ▶); data reduction: *SAINT*; program(s) used to solve structure: *SHELXS97* (Sheldrick, 2008 ▶); program(s) used to refine structure: *SHELXL97* (Sheldrick, 2008 ▶); molecular graphics: *XP* in *SHELXTL* (Sheldrick, 2008 ▶); software used to prepare material for publication: *XP* in *SHELXTL*.

## Supplementary Material

Crystal structure: contains datablocks global, I. DOI: 10.1107/S1600536808024537/bx2152sup1.cif

Structure factors: contains datablocks I. DOI: 10.1107/S1600536808024537/bx2152Isup2.hkl

Additional supplementary materials:  crystallographic information; 3D view; checkCIF report

## Figures and Tables

**Table:** 

Au1—N1	2.027 (3)
Au1—C17	2.105 (3)
Au1—C1	2.111 (3)
Au1—P1	2.3121 (9)
Au2—N2	2.041 (3)
Au2—C18	2.091 (3)
Au2—C34	2.113 (3)
Au2—P1	2.3180 (8)

**Table:** 

N1—Au1—C17	79.97 (12)
N1—Au1—C1	80.18 (12)
C17—Au1—C1	160.10 (13)
N1—Au1—P1	172.84 (8)
C17—Au1—P1	95.15 (9)
C1—Au1—P1	104.75 (10)
N2—Au2—C18	80.19 (12)
N2—Au2—C34	80.02 (12)
C18—Au2—C34	160.15 (13)
N2—Au2—P1	173.99 (8)
C18—Au2—P1	93.98 (9)
C34—Au2—P1	105.84 (9)

**Table 2:** Hydrogen-bond geometry (Å, °)

*D*—H⋯*A*	*D*—H	H⋯*A*	*D*⋯*A*	*D*—H⋯*A*
C21—H21⋯O3^i^	0.95	2.46	3.311 (7)	149
C36—H36⋯O2	0.95	2.57	3.372 (8)	143
C38—H38⋯O2^ii^	0.95	2.59	3.540 (5)	175
C43—H43⋯O1^iii^	0.95	2.59	3.517 (3)	165
C46—H46⋯N3^iv^	0.95	2.62	3.417 (4)	142
C48—H48*C*⋯O1^v^	0.98	2.54	3.423 (8)	150

Symmetry codes: (i) 
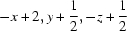
; (ii) 
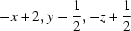
; (iii) 
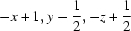
; (iv) 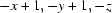; (v) 
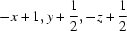
.

**Fig. 1.**
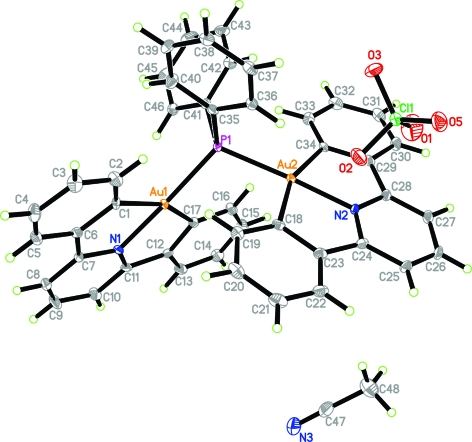
Molecular structure of the title compound showing the atom-numbering scheme and displacement ellipsoids drawn at the 40% probability level.

**Fig. 2.**
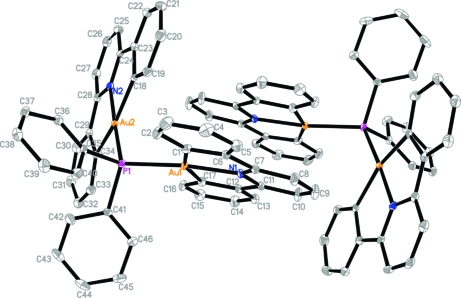
There is π-π interaction between between neighbouring molecules. The anions and solvent molecules are not shown, and H atoms have been omitted. Displacement ellipsoids are at the 40% probability level and Atoms without label are generated by the symmetry operation (1 - x, 1 - y, -z).

**Fig. 3.**
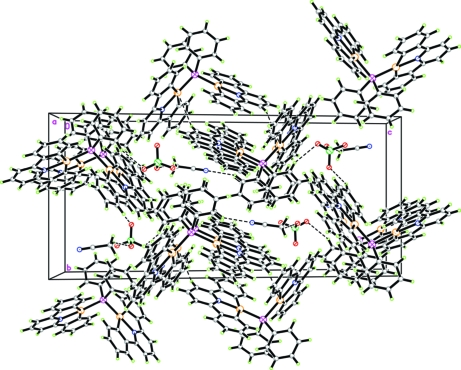
The crystal packing of the complex, viewed down the a axis, showing hydrogen bonds as dashed lines.

## supplementary crystallographic information

### Comment

Recently, extensive investigations on the biological properties of gold(III) have been reported (Kui *et al.*, 2006; Wong *et al.*, 1998). The stability of metal compounds is usually enhanced by multidentate chelating ligands. Li and his co-workers have reported multinuclear gold complex of 2,6-diphenyl-Pyridine ligand (dpp) to generate a planar gold(III) moiety (Li *et al.*, 2006). In this context, the title complex, (I), has been prepared and its crystal structure is reported here.

The title compound show gold(III) atoms bridged by diphenylphosphanide. Each Au atom is surrounded by one P atom from diphenylphosphanide ligand and two C atoms, one N atom from dpp in a square-planar geometry, the least-squares plane through Au^III^ and dpp has a mean deviation of 0.04 (3) Å. The intramolecular Au···Au contact of 3.788Å is beyond a normal range of metal-metal interactions for d^8^ metal ions (3.09–3.50 Å) (Yam *et al.*, 2002; Goshe *et al.*, 2003; Lu *et al.*, 2004). The Au–N(pyridyl) distances (2.027 (3), 2.041 (3) Å) are comparable to the related distances found in [Au(dpp)*L*]^n+^ analogues (1.94–2.06 Å), The Au–*C*(phenyl) distances (2.091 (3)–2.113 (3) Å) are comparable to the related distances found in [Au(dpp)*L*]^n+^ analogues (2.06–2.13 Å) (Li *et al.*, 2006). The interplanar separation between two neighbouring dpp molecules are 3.40 and 3.57Å (Fig. 2). The crystal packing shows weak intermolecular C-H..O and C-H···N hydrogen bonds with O atoms of perchlorate counter-ion and N atom of acetonitrile molecule respectively. (Fig. 3, Table 2).

### Experimental

A mixture of Au(dpp)Cl (0.092 g, 0.2 mmol) and diphenylphosphine (0.018 g, 0.1 mmol) in acetonitrile (30 ml) was stirred for 2 h. Excess LiClO_4_ was then added to yield a yellow precipitate, which was filtered, washed with diethyl ether. The precipitate was redissolve in acetonitrile. Yellow crystals suitable for X-ray diffraction were formed by vapour diffusion of diethyl ethyl ether into acetonitrile solution.

### Refinement

All hydrogen atoms were generated geometrically (C—H bond lengths of methyl group fixed at 0.98 Å, C—H bond lengths of pyridyl and phenyl fixed at 0.95 Å), assigned appropriated isotropic thermal parameters, *U*_iso_(H) = 1.2*U*_eq_(C).

### Crystal data

**Table:**

[Au_2_(C_17_H_11_N)_2_(C_12_H_10_P)]ClO_4_·C_2_H_3_N	*F*_000_ = 2264
*M**_r_* = 1178.14	*D*_x_ = 1.963 Mg m^−^^3^
Monoclinic, *P*2_1_/*c*	Mo *K*α radiation λ = 0.71070 Å
Hall symbol: -P 2ybc	Cell parameters from 13552 reflections
*a* = 10.0612 (16) Å	θ = 1.4–28.7º
*b* = 13.526 (2) Å	µ = 7.51 mm^−^^1^
*c* = 29.640 (5) Å	*T* = 113 (2) K
β = 98.828 (4)º	Block, yellow
*V* = 3985.9 (11) Å^3^	0.32 × 0.22 × 0.20 mm
*Z* = 4	

### Data collection

**Table:**

Bruker SMART CCD area-detector diffractometer	10221 independent reflections
Radiation source: fine-focus sealed tube	8923 reflections with *I* > 2σ(*I*)
Monochromator: graphite	*R*_int_ = 0.039
Detector resolution: 7.31 pixels mm^-1^	θ_max_ = 28.7º
*T* = 113(2) K	θ_min_ = 1.4º
φ and ω scans	*h* = −13→13
Absorption correction: multi-scan(SADABS; Sheldrick, 1996)	*k* = −18→17
*T*_min_ = 0.147, *T*_max_ = 0.221	*l* = −39→39
38570 measured reflections	

### Refinement

**Table:**

Refinement on *F*^2^	Secondary atom site location: difference Fourier map
Least-squares matrix: full	Hydrogen site location: inferred from neighbouring sites
*R*[*F*^2^ > 2σ(*F*^2^)] = 0.028	H-atom parameters constrained
*wR*(*F*^2^) = 0.057	*w* = 1/[σ^2^(*F*_o_^2^) + (0.0278*P*)^2^] where *P* = (*F*_o_^2^ + 2*F*_c_^2^)/3
*S* = 1.04	(Δ/σ)_max_ = 0.002
10221 reflections	Δρ_max_ = 1.52 e Å^−^^3^
534 parameters	Δρ_min_ = −1.55 e Å^−^^3^
Primary atom site location: structure-invariant direct methods	Extinction correction: none

### Special details

**Table:**

Geometry. All e.s.d.'s (except the e.s.d. in the dihedral angle between two l.s. planes) are estimated using the full covariance matrix. The cell e.s.d.'s are taken into account individually in the estimation of e.s.d.'s in distances, angles and torsion angles; correlations between e.s.d.'s in cell parameters are only used when they are defined by crystal symmetry. An approximate (isotropic) treatment of cell e.s.d.'s is used for estimating e.s.d.'s involving l.s. planes.
Refinement. Refinement of *F*^2^ against ALL reflections. The weighted *R*-factor *wR* and goodness of fit *S* are based on *F*^2^, conventional *R*-factors *R* are based on *F*, with *F* set to zero for negative *F*^2^. The threshold expression of *F*^2^ > σ(*F*^2^) is used only for calculating *R*-factors(gt) *etc*. and is not relevant to the choice of reflections for refinement. *R*-factors based on *F*^2^ are statistically about twice as large as those based on *F*, and *R*- factors based on ALL data will be even larger.

### Fractional atomic coordinates and isotropic or equivalent isotropic displacement parameters (Å^2^)

**Table:**

	*x*	*y*	*z*	*U*_iso_*/*U*_eq_	
Au1	0.638497 (12)	0.281287 (9)	0.032930 (4)	0.01055 (4)	
Au2	0.566168 (12)	0.331694 (9)	0.152497 (4)	0.00993 (4)	
Cl1	0.78527 (10)	0.29051 (6)	0.29597 (3)	0.01996 (19)	
P1	0.60967 (9)	0.21001 (6)	0.10167 (3)	0.01063 (17)	
O1	0.6459 (3)	0.3221 (3)	0.29088 (12)	0.0507 (9)	
O2	0.8447 (3)	0.3313 (2)	0.25911 (10)	0.0406 (8)	
O3	0.7868 (3)	0.18429 (18)	0.29417 (10)	0.0324 (7)	
O5	0.8547 (3)	0.3239 (2)	0.33921 (10)	0.0333 (7)	
N1	0.6406 (3)	0.33719 (18)	−0.03039 (9)	0.0119 (6)	
N2	0.5487 (3)	0.44270 (18)	0.19785 (9)	0.0125 (6)	
N3	0.6293 (4)	0.8322 (2)	0.06888 (13)	0.0353 (9)	
C1	0.8332 (3)	0.2378 (2)	0.02273 (12)	0.0134 (7)	
C2	0.9377 (4)	0.1917 (3)	0.05082 (13)	0.0196 (8)	
H2	0.9268	0.1750	0.0812	0.024*	
C3	1.0580 (4)	0.1693 (3)	0.03557 (14)	0.0234 (8)	
H3	1.1276	0.1372	0.0556	0.028*	
C4	1.0781 (4)	0.1929 (3)	−0.00813 (14)	0.0227 (8)	
H4	1.1602	0.1759	−0.0183	0.027*	
C5	0.9785 (4)	0.2413 (3)	−0.03691 (13)	0.0187 (8)	
H5	0.9924	0.2590	−0.0669	0.022*	
C6	0.8559 (3)	0.2646 (2)	−0.02188 (12)	0.0134 (7)	
C7	0.7489 (3)	0.3170 (2)	−0.05110 (12)	0.0131 (7)	
C8	0.7447 (4)	0.3461 (2)	−0.09612 (13)	0.0189 (8)	
H8	0.8185	0.3328	−0.1117	0.023*	
C9	0.6330 (4)	0.3944 (3)	−0.11806 (13)	0.0248 (9)	
H9	0.6298	0.4134	−0.1491	0.030*	
C10	0.5247 (4)	0.4158 (3)	−0.09585 (13)	0.0214 (8)	
H10	0.4492	0.4511	−0.1110	0.026*	
C11	0.5288 (3)	0.3846 (2)	−0.05116 (12)	0.0142 (7)	
C12	0.4238 (3)	0.3933 (2)	−0.02224 (11)	0.0127 (7)	
C13	0.3035 (4)	0.4442 (2)	−0.03724 (12)	0.0184 (7)	
H13	0.2895	0.4741	−0.0666	0.022*	
C14	0.2057 (4)	0.4508 (3)	−0.00958 (13)	0.0205 (8)	
H14	0.1246	0.4858	−0.0195	0.025*	
C15	0.2268 (4)	0.4059 (3)	0.03288 (13)	0.0207 (8)	
H15	0.1593	0.4100	0.0520	0.025*	
C16	0.3458 (4)	0.3546 (2)	0.04811 (12)	0.0173 (7)	
H16	0.3577	0.3238	0.0773	0.021*	
C17	0.4473 (3)	0.3480 (2)	0.02116 (12)	0.0134 (7)	
C18	0.7480 (3)	0.4054 (2)	0.15075 (11)	0.0134 (7)	
C19	0.8548 (3)	0.3858 (3)	0.12706 (12)	0.0159 (7)	
H19	0.8515	0.3290	0.1080	0.019*	
C20	0.9665 (4)	0.4483 (3)	0.13097 (13)	0.0233 (8)	
H20	1.0380	0.4332	0.1146	0.028*	
C21	0.9750 (4)	0.5317 (3)	0.15812 (14)	0.0232 (8)	
H21	1.0506	0.5743	0.1599	0.028*	
C22	0.8716 (4)	0.5523 (3)	0.18275 (12)	0.0211 (8)	
H22	0.8770	0.6089	0.2019	0.025*	
C23	0.7597 (3)	0.4903 (2)	0.17957 (12)	0.0158 (7)	
C24	0.6487 (4)	0.5093 (2)	0.20564 (12)	0.0154 (7)	
C25	0.6358 (4)	0.5876 (2)	0.23508 (12)	0.0205 (8)	
H25	0.7051	0.6355	0.2416	0.025*	
C26	0.5196 (4)	0.5942 (2)	0.25476 (12)	0.0215 (8)	
H26	0.5077	0.6487	0.2739	0.026*	
C27	0.4211 (4)	0.5227 (2)	0.24684 (12)	0.0188 (8)	
H27	0.3433	0.5269	0.2613	0.023*	
C28	0.4362 (3)	0.4449 (2)	0.21773 (11)	0.0135 (7)	
C29	0.3418 (3)	0.3654 (2)	0.20285 (12)	0.0145 (7)	
C30	0.2188 (4)	0.3568 (3)	0.22005 (13)	0.0190 (8)	
H30	0.1987	0.4011	0.2429	0.023*	
C31	0.1278 (4)	0.2837 (3)	0.20352 (13)	0.0207 (8)	
H31	0.0463	0.2762	0.2157	0.025*	
C32	0.1563 (4)	0.2214 (3)	0.16901 (13)	0.0202 (8)	
H32	0.0926	0.1727	0.1569	0.024*	
C33	0.2777 (3)	0.2295 (2)	0.15193 (12)	0.0171 (7)	
H33	0.2949	0.1864	0.1282	0.021*	
C34	0.3738 (3)	0.2993 (2)	0.16887 (11)	0.0124 (7)	
C35	0.7453 (3)	0.1350 (2)	0.13295 (11)	0.0128 (7)	
C36	0.7981 (3)	0.1545 (2)	0.17805 (12)	0.0139 (7)	
H36	0.7716	0.2125	0.1924	0.017*	
C37	0.8898 (3)	0.0893 (2)	0.20256 (12)	0.0172 (7)	
H37	0.9238	0.1024	0.2337	0.021*	
C38	0.9313 (3)	0.0060 (3)	0.18167 (13)	0.0184 (8)	
H38	0.9949	−0.0376	0.1982	0.022*	
C39	0.8799 (4)	−0.0139 (3)	0.13654 (13)	0.0208 (8)	
H39	0.9095	−0.0707	0.1220	0.025*	
C40	0.7854 (4)	0.0489 (3)	0.11244 (13)	0.0204 (8)	
H40	0.7476	0.0334	0.0819	0.024*	
C41	0.4844 (3)	0.1125 (2)	0.08729 (11)	0.0122 (7)	
C42	0.4565 (4)	0.0507 (2)	0.12251 (13)	0.0215 (8)	
H42	0.5013	0.0611	0.1528	0.026*	
C43	0.3644 (4)	−0.0252 (3)	0.11367 (15)	0.0268 (9)	
H43	0.3430	−0.0650	0.1380	0.032*	
C44	0.3032 (4)	−0.0431 (3)	0.06923 (16)	0.0282 (10)	
H44	0.2401	−0.0954	0.0631	0.034*	
C45	0.3338 (4)	0.0151 (3)	0.03385 (14)	0.0242 (8)	
H45	0.2940	0.0011	0.0034	0.029*	
C46	0.4236 (3)	0.0948 (2)	0.04281 (12)	0.0155 (7)	
H46	0.4424	0.1361	0.0186	0.019*	
C47	0.6302 (4)	0.8197 (3)	0.10658 (16)	0.0301 (10)	
C48	0.6321 (5)	0.8047 (4)	0.15590 (16)	0.0448 (12)	
H48A	0.6670	0.8642	0.1725	0.067*	
H48B	0.6899	0.7482	0.1662	0.067*	
H48C	0.5405	0.7916	0.1618	0.067*	

### Atomic displacement parameters (Å^2^)

**Table:**

	*U*^11^	*U*^22^	*U*^33^	*U*^12^	*U*^13^	*U*^23^
Au1	0.01160 (7)	0.01217 (6)	0.00809 (6)	0.00003 (5)	0.00215 (5)	0.00087 (4)
Au2	0.01163 (7)	0.00982 (6)	0.00834 (6)	−0.00009 (5)	0.00155 (5)	−0.00021 (4)
Cl1	0.0299 (5)	0.0169 (4)	0.0141 (4)	−0.0020 (4)	0.0064 (4)	−0.0008 (3)
P1	0.0135 (4)	0.0105 (4)	0.0080 (4)	0.0006 (3)	0.0019 (3)	−0.0002 (3)
O1	0.036 (2)	0.065 (2)	0.050 (2)	0.0258 (17)	0.0025 (17)	−0.0007 (18)
O2	0.068 (2)	0.0353 (17)	0.0223 (17)	−0.0232 (16)	0.0181 (17)	−0.0033 (13)
O3	0.052 (2)	0.0138 (13)	0.0306 (18)	−0.0011 (13)	0.0035 (15)	−0.0016 (11)
O5	0.0417 (19)	0.0377 (17)	0.0206 (16)	−0.0132 (14)	0.0046 (14)	−0.0053 (13)
N1	0.0158 (15)	0.0110 (13)	0.0094 (14)	−0.0019 (11)	0.0036 (12)	0.0016 (10)
N2	0.0196 (15)	0.0096 (12)	0.0082 (14)	0.0016 (12)	0.0016 (12)	−0.0013 (10)
N3	0.052 (3)	0.0319 (19)	0.023 (2)	−0.0020 (18)	0.0084 (19)	−0.0059 (15)
C1	0.0123 (17)	0.0161 (16)	0.0127 (17)	−0.0003 (13)	0.0049 (14)	−0.0046 (13)
C2	0.0166 (19)	0.0259 (19)	0.0154 (19)	0.0015 (15)	−0.0007 (15)	0.0031 (15)
C3	0.018 (2)	0.030 (2)	0.021 (2)	0.0035 (16)	−0.0010 (16)	−0.0006 (16)
C4	0.0126 (19)	0.032 (2)	0.024 (2)	0.0026 (16)	0.0060 (16)	−0.0055 (16)
C5	0.0176 (19)	0.0222 (18)	0.0174 (19)	−0.0019 (15)	0.0063 (15)	−0.0009 (14)
C6	0.0142 (18)	0.0123 (15)	0.0136 (18)	−0.0023 (13)	0.0022 (14)	−0.0010 (12)
C7	0.0164 (18)	0.0111 (15)	0.0122 (17)	−0.0015 (13)	0.0035 (14)	−0.0016 (12)
C8	0.026 (2)	0.0181 (17)	0.0140 (19)	0.0015 (15)	0.0083 (16)	0.0009 (14)
C9	0.032 (2)	0.030 (2)	0.014 (2)	0.0038 (18)	0.0091 (17)	0.0079 (16)
C10	0.029 (2)	0.0221 (18)	0.0139 (19)	0.0071 (16)	0.0040 (16)	0.0076 (14)
C11	0.0203 (18)	0.0089 (15)	0.0127 (18)	−0.0018 (13)	0.0004 (14)	0.0021 (12)
C12	0.0162 (18)	0.0103 (15)	0.0111 (17)	−0.0010 (13)	0.0003 (14)	0.0001 (12)
C13	0.0211 (19)	0.0169 (16)	0.0164 (19)	−0.0007 (15)	0.0002 (15)	0.0039 (14)
C14	0.0180 (19)	0.0204 (18)	0.023 (2)	0.0064 (15)	0.0015 (16)	0.0034 (15)
C15	0.0155 (19)	0.0259 (19)	0.022 (2)	0.0023 (15)	0.0072 (16)	−0.0017 (15)
C16	0.0186 (19)	0.0216 (17)	0.0112 (18)	0.0016 (15)	0.0008 (14)	0.0029 (13)
C17	0.0134 (17)	0.0121 (15)	0.0151 (18)	0.0019 (13)	0.0034 (14)	0.0002 (13)
C18	0.0132 (17)	0.0153 (16)	0.0103 (17)	−0.0020 (13)	−0.0026 (13)	0.0045 (13)
C19	0.0131 (17)	0.0194 (17)	0.0147 (18)	0.0002 (14)	0.0001 (14)	0.0009 (14)
C20	0.018 (2)	0.032 (2)	0.020 (2)	−0.0012 (17)	0.0048 (16)	0.0053 (16)
C21	0.0145 (19)	0.027 (2)	0.027 (2)	−0.0088 (16)	−0.0008 (16)	0.0053 (16)
C22	0.028 (2)	0.0164 (17)	0.017 (2)	−0.0059 (16)	−0.0027 (16)	0.0020 (14)
C23	0.0172 (18)	0.0145 (16)	0.0145 (18)	−0.0003 (14)	−0.0013 (14)	0.0035 (13)
C24	0.0219 (19)	0.0123 (15)	0.0098 (17)	−0.0021 (14)	−0.0045 (14)	0.0009 (12)
C25	0.031 (2)	0.0137 (16)	0.0152 (19)	−0.0057 (15)	−0.0004 (16)	−0.0037 (13)
C26	0.039 (2)	0.0150 (17)	0.0103 (18)	0.0040 (16)	0.0026 (17)	−0.0039 (13)
C27	0.026 (2)	0.0168 (17)	0.0143 (19)	0.0075 (15)	0.0049 (16)	−0.0001 (13)
C28	0.0166 (18)	0.0138 (15)	0.0100 (17)	0.0046 (14)	0.0016 (14)	0.0006 (13)
C29	0.0154 (18)	0.0160 (16)	0.0116 (17)	0.0027 (14)	0.0008 (14)	0.0036 (13)
C30	0.0187 (19)	0.0213 (18)	0.019 (2)	0.0076 (15)	0.0076 (16)	0.0000 (14)
C31	0.0130 (18)	0.029 (2)	0.021 (2)	0.0020 (15)	0.0049 (15)	0.0052 (16)
C32	0.0130 (18)	0.0248 (19)	0.022 (2)	−0.0003 (15)	0.0016 (15)	0.0050 (15)
C33	0.0154 (18)	0.0188 (17)	0.0165 (19)	0.0022 (14)	0.0001 (15)	−0.0004 (14)
C34	0.0146 (17)	0.0144 (15)	0.0081 (16)	0.0001 (13)	0.0019 (13)	0.0029 (12)
C35	0.0154 (17)	0.0130 (15)	0.0104 (17)	0.0010 (13)	0.0033 (14)	0.0030 (12)
C36	0.0113 (17)	0.0159 (16)	0.0151 (18)	−0.0001 (13)	0.0034 (14)	0.0010 (13)
C37	0.0122 (17)	0.0244 (18)	0.0143 (19)	−0.0003 (14)	−0.0001 (14)	0.0054 (14)
C38	0.0141 (18)	0.0191 (17)	0.023 (2)	0.0032 (14)	0.0062 (15)	0.0063 (14)
C39	0.025 (2)	0.0153 (17)	0.024 (2)	0.0075 (15)	0.0075 (17)	−0.0007 (14)
C40	0.023 (2)	0.0201 (18)	0.017 (2)	0.0035 (15)	−0.0011 (15)	−0.0027 (14)
C41	0.0136 (17)	0.0104 (15)	0.0131 (17)	−0.0016 (13)	0.0034 (14)	−0.0030 (12)
C42	0.029 (2)	0.0169 (17)	0.020 (2)	−0.0023 (16)	0.0071 (17)	0.0006 (15)
C43	0.031 (2)	0.0155 (18)	0.037 (3)	−0.0063 (16)	0.015 (2)	0.0023 (16)
C44	0.022 (2)	0.0159 (18)	0.048 (3)	−0.0069 (16)	0.009 (2)	−0.0033 (17)
C45	0.020 (2)	0.0223 (19)	0.028 (2)	−0.0004 (16)	−0.0060 (16)	−0.0089 (16)
C46	0.0137 (18)	0.0177 (16)	0.0149 (18)	0.0008 (14)	0.0019 (14)	−0.0018 (13)
C47	0.040 (3)	0.0199 (19)	0.031 (3)	0.0015 (18)	0.006 (2)	−0.0050 (17)
C48	0.062 (3)	0.046 (3)	0.027 (3)	−0.001 (3)	0.008 (2)	0.008 (2)

### Geometric parameters (Å, °)

**Table:**

Au1—N1	2.027 (3)	C20—C21	1.380 (5)
Au1—C17	2.105 (3)	C20—H20	0.9500
Au1—C1	2.111 (3)	C21—C22	1.388 (5)
Au1—P1	2.3121 (9)	C21—H21	0.9500
Au2—N2	2.041 (3)	C22—C23	1.395 (5)
Au2—C18	2.091 (3)	C22—H22	0.9500
Au2—C34	2.113 (3)	C23—C24	1.474 (5)
Au2—P1	2.3180 (8)	C24—C25	1.391 (4)
Cl1—O2	1.434 (3)	C25—C26	1.387 (5)
Cl1—O5	1.436 (3)	C25—H25	0.9500
Cl1—O3	1.438 (3)	C26—C27	1.378 (5)
Cl1—O1	1.451 (3)	C26—H26	0.9500
P1—C41	1.829 (3)	C27—C28	1.384 (4)
P1—C35	1.833 (3)	C27—H27	0.9500
N1—C11	1.357 (4)	C28—C29	1.458 (5)
N1—C7	1.358 (4)	C29—C30	1.413 (4)
N2—C24	1.344 (4)	C29—C34	1.421 (4)
N2—C28	1.354 (4)	C30—C31	1.386 (5)
N3—C47	1.129 (5)	C30—H30	0.9500
C1—C2	1.385 (5)	C31—C32	1.389 (5)
C1—C6	1.423 (5)	C31—H31	0.9500
C2—C3	1.389 (5)	C32—C33	1.396 (5)
C2—H2	0.9500	C32—H32	0.9500
C3—C4	1.379 (5)	C33—C34	1.389 (5)
C3—H3	0.9500	C33—H33	0.9500
C4—C5	1.379 (5)	C35—C36	1.386 (5)
C4—H4	0.9500	C35—C40	1.402 (4)
C5—C6	1.409 (4)	C36—C37	1.397 (5)
C5—H5	0.9500	C36—H36	0.9500
C6—C7	1.457 (5)	C37—C38	1.382 (5)
C7—C8	1.386 (5)	C37—H37	0.9500
C8—C9	1.374 (5)	C38—C39	1.385 (5)
C8—H8	0.9500	C38—H38	0.9500
C9—C10	1.387 (5)	C39—C40	1.389 (5)
C9—H9	0.9500	C39—H39	0.9500
C10—C11	1.384 (5)	C40—H40	0.9500
C10—H10	0.9500	C41—C46	1.387 (5)
C11—C12	1.464 (4)	C41—C42	1.399 (4)
C12—C13	1.404 (5)	C42—C43	1.382 (5)
C12—C17	1.412 (4)	C42—H42	0.9500
C13—C14	1.377 (5)	C43—C44	1.387 (6)
C13—H13	0.9500	C43—H43	0.9500
C14—C15	1.384 (5)	C44—C45	1.383 (5)
C14—H14	0.9500	C44—H44	0.9500
C15—C16	1.397 (5)	C45—C46	1.405 (5)
C15—H15	0.9500	C45—H45	0.9500
C16—C17	1.392 (4)	C46—H46	0.9500
C16—H16	0.9500	C47—C48	1.473 (6)
C18—C19	1.397 (4)	C48—H48A	0.9800
C18—C23	1.425 (5)	C48—H48B	0.9800
C19—C20	1.397 (5)	C48—H48C	0.9800
C19—H19	0.9500		
			
N1—Au1—C17	79.97 (12)	C21—C20—H20	119.3
N1—Au1—C1	80.18 (12)	C19—C20—H20	119.3
C17—Au1—C1	160.10 (13)	C20—C21—C22	119.1 (3)
N1—Au1—P1	172.84 (8)	C20—C21—H21	120.5
C17—Au1—P1	95.15 (9)	C22—C21—H21	120.5
C1—Au1—P1	104.75 (10)	C21—C22—C23	120.4 (3)
N2—Au2—C18	80.19 (12)	C21—C22—H22	119.8
N2—Au2—C34	80.02 (12)	C23—C22—H22	119.8
C18—Au2—C34	160.15 (13)	C22—C23—C18	121.1 (3)
N2—Au2—P1	173.99 (8)	C22—C23—C24	121.9 (3)
C18—Au2—P1	93.98 (9)	C18—C23—C24	117.1 (3)
C34—Au2—P1	105.84 (9)	N2—C24—C25	118.6 (3)
O2—Cl1—O5	110.88 (18)	N2—C24—C23	113.6 (3)
O2—Cl1—O3	110.39 (17)	C25—C24—C23	127.8 (3)
O5—Cl1—O3	109.96 (18)	C26—C25—C24	118.6 (3)
O2—Cl1—O1	108.4 (2)	C26—C25—H25	120.7
O5—Cl1—O1	109.28 (19)	C24—C25—H25	120.7
O3—Cl1—O1	107.8 (2)	C27—C26—C25	120.9 (3)
C41—P1—C35	98.71 (15)	C27—C26—H26	119.6
C41—P1—Au1	105.69 (11)	C25—C26—H26	119.6
C35—P1—Au1	119.48 (10)	C26—C27—C28	119.7 (3)
C41—P1—Au2	118.14 (10)	C26—C27—H27	120.2
C35—P1—Au2	105.32 (11)	C28—C27—H27	120.2
Au1—P1—Au2	109.78 (3)	N2—C28—C27	117.8 (3)
C11—N1—C7	123.8 (3)	N2—C28—C29	113.7 (3)
C11—N1—Au1	118.1 (2)	C27—C28—C29	128.4 (3)
C7—N1—Au1	117.8 (2)	C30—C29—C34	120.7 (3)
C24—N2—C28	124.4 (3)	C30—C29—C28	121.2 (3)
C24—N2—Au2	117.9 (2)	C34—C29—C28	118.1 (3)
C28—N2—Au2	117.7 (2)	C31—C30—C29	119.9 (3)
C2—C1—C6	117.3 (3)	C31—C30—H30	120.0
C2—C1—Au1	132.4 (3)	C29—C30—H30	120.0
C6—C1—Au1	110.3 (2)	C30—C31—C32	119.6 (3)
C1—C2—C3	121.4 (3)	C30—C31—H31	120.2
C1—C2—H2	119.3	C32—C31—H31	120.2
C3—C2—H2	119.3	C31—C32—C33	120.7 (3)
C4—C3—C2	121.1 (4)	C31—C32—H32	119.7
C4—C3—H3	119.5	C33—C32—H32	119.7
C2—C3—H3	119.5	C34—C33—C32	121.5 (3)
C5—C4—C3	119.6 (3)	C34—C33—H33	119.3
C5—C4—H4	120.2	C32—C33—H33	119.3
C3—C4—H4	120.2	C33—C34—C29	117.6 (3)
C4—C5—C6	120.0 (3)	C33—C34—Au2	132.0 (2)
C4—C5—H5	120.0	C29—C34—Au2	110.3 (2)
C6—C5—H5	120.0	C36—C35—C40	118.9 (3)
C5—C6—C1	120.6 (3)	C36—C35—P1	121.9 (2)
C5—C6—C7	121.5 (3)	C40—C35—P1	118.8 (3)
C1—C6—C7	117.8 (3)	C35—C36—C37	120.5 (3)
N1—C7—C8	118.0 (3)	C35—C36—H36	119.8
N1—C7—C6	113.6 (3)	C37—C36—H36	119.8
C8—C7—C6	128.4 (3)	C38—C37—C36	120.2 (3)
C9—C8—C7	119.5 (3)	C38—C37—H37	119.9
C9—C8—H8	120.2	C36—C37—H37	119.9
C7—C8—H8	120.2	C37—C38—C39	119.9 (3)
C8—C9—C10	121.3 (3)	C37—C38—H38	120.1
C8—C9—H9	119.3	C39—C38—H38	120.1
C10—C9—H9	119.3	C38—C39—C40	120.2 (3)
C11—C10—C9	118.7 (3)	C38—C39—H39	119.9
C11—C10—H10	120.7	C40—C39—H39	119.9
C9—C10—H10	120.7	C39—C40—C35	120.4 (3)
N1—C11—C10	118.6 (3)	C39—C40—H40	119.8
N1—C11—C12	113.2 (3)	C35—C40—H40	119.8
C10—C11—C12	128.1 (3)	C46—C41—C42	119.8 (3)
C13—C12—C17	121.2 (3)	C46—C41—P1	122.3 (3)
C13—C12—C11	121.2 (3)	C42—C41—P1	117.8 (3)
C17—C12—C11	117.6 (3)	C43—C42—C41	120.6 (4)
C14—C13—C12	120.2 (3)	C43—C42—H42	119.7
C14—C13—H13	119.9	C41—C42—H42	119.7
C12—C13—H13	119.9	C42—C43—C44	119.8 (4)
C13—C14—C15	119.2 (3)	C42—C43—H43	120.1
C13—C14—H14	120.4	C44—C43—H43	120.1
C15—C14—H14	120.4	C45—C44—C43	120.2 (3)
C14—C15—C16	121.1 (3)	C45—C44—H44	119.9
C14—C15—H15	119.5	C43—C44—H44	119.9
C16—C15—H15	119.5	C44—C45—C46	120.3 (4)
C17—C16—C15	120.9 (3)	C44—C45—H45	119.8
C17—C16—H16	119.5	C46—C45—H45	119.8
C15—C16—H16	119.5	C41—C46—C45	119.3 (3)
C16—C17—C12	117.4 (3)	C41—C46—H46	120.3
C16—C17—Au1	131.6 (3)	C45—C46—H46	120.3
C12—C17—Au1	111.0 (2)	N3—C47—C48	179.2 (5)
C19—C18—C23	117.1 (3)	C47—C48—H48A	109.5
C19—C18—Au2	131.7 (3)	C47—C48—H48B	109.5
C23—C18—Au2	111.2 (2)	H48A—C48—H48B	109.5
C18—C19—C20	120.9 (3)	C47—C48—H48C	109.5
C18—C19—H19	119.5	H48A—C48—H48C	109.5
C20—C19—H19	119.5	H48B—C48—H48C	109.5
C21—C20—C19	121.4 (3)		
			
C17—Au1—P1—C41	69.65 (14)	C34—Au2—C18—C23	3.3 (5)
C1—Au1—P1—C41	−109.99 (14)	P1—Au2—C18—C23	179.9 (2)
C17—Au1—P1—C35	179.50 (16)	C23—C18—C19—C20	−1.3 (5)
C1—Au1—P1—C35	−0.14 (16)	Au2—C18—C19—C20	178.8 (3)
C17—Au1—P1—Au2	−58.78 (10)	C18—C19—C20—C21	−0.2 (6)
C1—Au1—P1—Au2	121.58 (10)	C19—C20—C21—C22	1.4 (6)
C18—Au2—P1—C41	179.02 (15)	C20—C21—C22—C23	−0.9 (6)
C34—Au2—P1—C41	−2.15 (16)	C21—C22—C23—C18	−0.7 (5)
C18—Au2—P1—C35	70.08 (14)	C21—C22—C23—C24	179.3 (3)
C34—Au2—P1—C35	−111.09 (14)	C19—C18—C23—C22	1.8 (5)
C18—Au2—P1—Au1	−59.77 (9)	Au2—C18—C23—C22	−178.3 (3)
C34—Au2—P1—Au1	119.07 (10)	C19—C18—C23—C24	−178.2 (3)
C17—Au1—N1—C11	−2.2 (2)	Au2—C18—C23—C24	1.7 (4)
C1—Au1—N1—C11	179.3 (3)	C28—N2—C24—C25	−1.7 (5)
C17—Au1—N1—C7	−176.2 (3)	Au2—N2—C24—C25	177.5 (2)
C1—Au1—N1—C7	5.3 (2)	C28—N2—C24—C23	−179.8 (3)
C18—Au2—N2—C24	1.2 (2)	Au2—N2—C24—C23	−0.6 (4)
C34—Au2—N2—C24	−177.2 (3)	C22—C23—C24—N2	179.2 (3)
C18—Au2—N2—C28	−179.5 (3)	C18—C23—C24—N2	−0.8 (4)
C34—Au2—N2—C28	2.1 (2)	C22—C23—C24—C25	1.3 (6)
N1—Au1—C1—C2	175.4 (4)	C18—C23—C24—C25	−178.7 (3)
C17—Au1—C1—C2	171.1 (3)	N2—C24—C25—C26	−0.7 (5)
P1—Au1—C1—C2	−9.9 (4)	C23—C24—C25—C26	177.0 (3)
N1—Au1—C1—C6	−3.4 (2)	C24—C25—C26—C27	2.6 (5)
C17—Au1—C1—C6	−7.7 (5)	C25—C26—C27—C28	−2.1 (5)
P1—Au1—C1—C6	171.3 (2)	C24—N2—C28—C27	2.2 (5)
C6—C1—C2—C3	−2.3 (5)	Au2—N2—C28—C27	−177.0 (2)
Au1—C1—C2—C3	179.0 (3)	C24—N2—C28—C29	178.8 (3)
C1—C2—C3—C4	0.5 (6)	Au2—N2—C28—C29	−0.4 (4)
C2—C3—C4—C5	1.3 (6)	C26—C27—C28—N2	−0.2 (5)
C3—C4—C5—C6	−1.3 (6)	C26—C27—C28—C29	−176.3 (3)
C4—C5—C6—C1	−0.6 (5)	N2—C28—C29—C30	−179.8 (3)
C4—C5—C6—C7	179.2 (3)	C27—C28—C29—C30	−3.6 (6)
C2—C1—C6—C5	2.3 (5)	N2—C28—C29—C34	−2.7 (4)
Au1—C1—C6—C5	−178.7 (3)	C27—C28—C29—C34	173.5 (3)
C2—C1—C6—C7	−177.5 (3)	C34—C29—C30—C31	−0.5 (5)
Au1—C1—C6—C7	1.5 (4)	C28—C29—C30—C31	176.6 (3)
C11—N1—C7—C8	−0.3 (5)	C29—C30—C31—C32	−2.1 (6)
Au1—N1—C7—C8	173.4 (2)	C30—C31—C32—C33	2.2 (6)
C11—N1—C7—C6	−179.5 (3)	C31—C32—C33—C34	0.4 (6)
Au1—N1—C7—C6	−5.8 (4)	C32—C33—C34—C29	−2.9 (5)
C5—C6—C7—N1	−177.2 (3)	C32—C33—C34—Au2	179.1 (3)
C1—C6—C7—N1	2.6 (4)	C30—C29—C34—C33	2.9 (5)
C5—C6—C7—C8	3.8 (5)	C28—C29—C34—C33	−174.2 (3)
C1—C6—C7—C8	−176.5 (3)	C30—C29—C34—Au2	−178.7 (3)
N1—C7—C8—C9	0.3 (5)	C28—C29—C34—Au2	4.2 (4)
C6—C7—C8—C9	179.4 (3)	N2—Au2—C34—C33	174.9 (3)
C7—C8—C9—C10	0.9 (6)	C18—Au2—C34—C33	170.1 (3)
C8—C9—C10—C11	−2.2 (6)	P1—Au2—C34—C33	−6.5 (3)
C7—N1—C11—C10	−1.0 (5)	N2—Au2—C34—C29	−3.3 (2)
Au1—N1—C11—C10	−174.6 (2)	C18—Au2—C34—C29	−8.1 (5)
C7—N1—C11—C12	177.3 (3)	P1—Au2—C34—C29	175.4 (2)
Au1—N1—C11—C12	3.7 (4)	C41—P1—C35—C36	−120.8 (3)
C9—C10—C11—N1	2.2 (5)	Au1—P1—C35—C36	125.5 (2)
C9—C10—C11—C12	−175.8 (3)	Au2—P1—C35—C36	1.6 (3)
N1—C11—C12—C13	177.0 (3)	C41—P1—C35—C40	51.5 (3)
C10—C11—C12—C13	−4.9 (6)	Au1—P1—C35—C40	−62.2 (3)
N1—C11—C12—C17	−3.5 (4)	Au2—P1—C35—C40	173.9 (2)
C10—C11—C12—C17	174.6 (3)	C40—C35—C36—C37	0.1 (5)
C17—C12—C13—C14	0.1 (5)	P1—C35—C36—C37	172.4 (2)
C11—C12—C13—C14	179.6 (3)	C35—C36—C37—C38	1.5 (5)
C12—C13—C14—C15	−0.7 (5)	C36—C37—C38—C39	−1.1 (5)
C13—C14—C15—C16	0.4 (6)	C37—C38—C39—C40	−1.0 (5)
C14—C15—C16—C17	0.6 (6)	C38—C39—C40—C35	2.7 (5)
C15—C16—C17—C12	−1.2 (5)	C36—C35—C40—C39	−2.2 (5)
C15—C16—C17—Au1	178.4 (3)	P1—C35—C40—C39	−174.7 (3)
C13—C12—C17—C16	0.9 (5)	C35—P1—C41—C46	−125.9 (3)
C11—C12—C17—C16	−178.6 (3)	Au1—P1—C41—C46	−1.8 (3)
C13—C12—C17—Au1	−178.8 (3)	Au2—P1—C41—C46	121.4 (3)
C11—C12—C17—Au1	1.7 (4)	C35—P1—C41—C42	50.2 (3)
N1—Au1—C17—C16	−179.4 (3)	Au1—P1—C41—C42	174.3 (2)
C1—Au1—C17—C16	−175.2 (3)	Au2—P1—C41—C42	−62.4 (3)
P1—Au1—C17—C16	5.8 (3)	C46—C41—C42—C43	−2.8 (5)
N1—Au1—C17—C12	0.2 (2)	P1—C41—C42—C43	−179.0 (3)
C1—Au1—C17—C12	4.4 (5)	C41—C42—C43—C44	2.8 (5)
P1—Au1—C17—C12	−174.6 (2)	C42—C43—C44—C45	−0.3 (6)
N2—Au2—C18—C19	178.4 (3)	C43—C44—C45—C46	−2.2 (6)
C34—Au2—C18—C19	−176.8 (3)	C42—C41—C46—C45	0.4 (5)
P1—Au2—C18—C19	−0.1 (3)	P1—C41—C46—C45	176.4 (2)
N2—Au2—C18—C23	−1.5 (2)	C44—C45—C46—C41	2.1 (5)

### Hydrogen-bond geometry (Å, °)

**Table:**

*D*—H···*A*	*D*—H	H···*A*	*D*···*A*	*D*—H···*A*
C21—H21···O3^i^	0.95	2.46	3.311 (7)	149
C36—H36···O2	0.95	2.57	3.372 (8)	143
C38—H38···O2^ii^	0.95	2.59	3.540 (5)	175
C43—H43···O1^iii^	0.95	2.59	3.517 (3)	165
C46—H46···N3^iv^	0.95	2.62	3.417 (4)	142
C48—H48C···O1^v^	0.98	2.54	3.423 (8)	150

Symmetry codes: (i) −*x*+2, *y*+1/2, −*z*+1/2; (ii) −*x*+2, *y*−1/2, −*z*+1/2; (iii) −*x*+1, *y*−1/2, −*z*+1/2; (iv) −*x*+1, −*y*+1, −*z*; (v) −*x*+1, *y*+1/2, −*z*+1/2.
